# Confounding factors in the diagnosis and clinical course of rare congenital hemolytic anemias

**DOI:** 10.1186/s13023-021-02036-4

**Published:** 2021-10-09

**Authors:** Bruno Fattizzo, Juri Alessandro Giannotta, Nicola Cecchi, Wilma Barcellini

**Affiliations:** 1 Hematology Unit, Fondazione IRCCS Ca’ Granda Ospedale Maggiore Policlinico, Milan, Italy; 2 Department of Oncology and Oncohematology, University of Milan, Milan, Italy

**Keywords:** Congenital hemolytic anemias, Iron overload, Splenectomy, Molecular studies, Differential diagnosis

## Abstract

Congenital hemolytic anemias (CHAs) comprise defects of the erythrocyte membrane proteins and of red blood cell enzymes metabolism, along with alterations of erythropoiesis. These rare and heterogeneous conditions may generate several difficulties from the diagnostic point of view. Membrane defects include hereditary spherocytosis and elliptocytosis, and the group of hereditary stomatocytosis; glucose-6-phosphate dehydrogenase and pyruvate kinase, are the most common enzyme deficiencies. Among ultra-rare forms, it is worth reminding other enzyme defects (glucosephosphate isomerase, phosphofructokinase, adenylate kinase, triosephosphate isomerase, phosphoglycerate kinase, hexokinase, and pyrimidine 5′-nucleotidase), and congenital dyserythropoietic anemias. Family history, clinical findings (anemia, hemolysis, splenomegaly, gallstones, and iron overload), red cells morphology, and biochemical tests are well recognized diagnostic tools. Molecular findings are increasingly used, particularly in recessive and de novo cases, and may be fundamental in unraveling the diagnosis. Notably, several confounders may further challenge the diagnostic workup, including concomitant blood loss, nutrients deficiency, alterations of hemolytic markers due to other causes (alloimmunization, infectious agents, rare metabolic disorders), coexistence of other hemolytic disorders (autoimmune hemolytic anemia, paroxysmal nocturnal hemoglobinuria, etc.). Additional factors to be considered are the possible association with bone marrow, renal or hepatic diseases, other causes of iron overload (hereditary hemochromatosis, hemoglobinopathies, metabolic diseases), and the presence of extra-hematological signs/symptoms. In this review we provide some instructive clinical vignettes that highlight the difficulties and confounders encountered in the diagnosis and clinical management of CHAs.

## Introduction

**Fig. 1:**
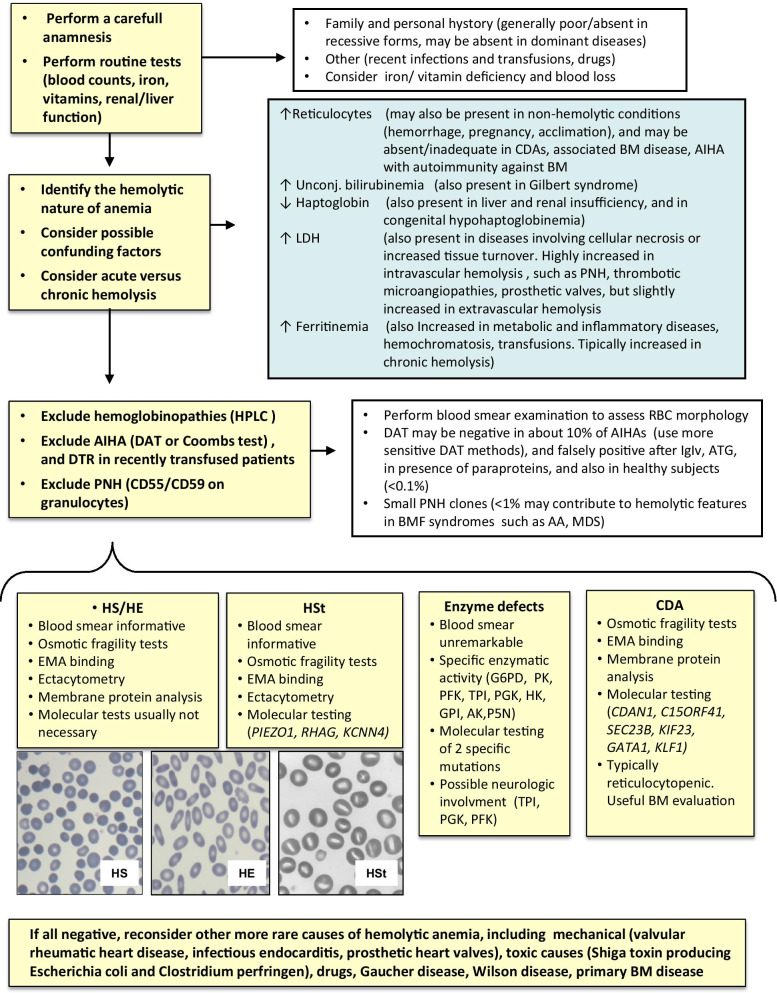
Diagnostic flow chart and possible confounders in congenital hemolytic anemias (CHAs). RBC: red blood cell, DAT: direct antiglobulin test, HPLC: high performance liquid chromatography, EMA-binding: eosin-5′-maleimide-labeled RBC by flow cytometric analysis, HS: hereditary spherocytosis, HE: hereditary elliptocytosis, HSt: hereditary stomatocytosis, CDA: congenital dyserythropoietic anemia, G6PD: glucose-6-phosphate dehydrogenase, PK: pyruvate kinase, GPI: glucose phosphate isomerise, PFK: phosphofructokinase, TPI: triose phosphate isomerase, PGK: phosphoglycerate kinase, HK: hexokinase, AK: adenylate kinase, P5N: pyrimidine 5'-nucleotidase deficiency. AIHA: autoimmune hemolytic anemia; DTR, delayed transfusion reaction, BM: bone marrow, PNH paroxysmal nocturnal hemoglobinuria, BMF bone marrow failure, AA aplastic anemia, MDS myelodysplastic syndrome

Congenital hemolytic anemias (CHAs) are rare diseases that comprise defects of the erythrocyte membrane proteins and of red blood cell (RBC) enzymes metabolism, along with alterations of erythropoiesis. These are very heterogeneous conditions both clinically and regarding genetic inheritance, arising several concerns from the diagnostic point of view. Clinically, CHAs are characterized by variable degree of anemia (from fully compensated to intra-uterine death) and hemolysis, increased erythrocyte turnover, splenomegaly, jaundice, biliary lithiasis, and iron overload. Beyond the pivotal importance of red cells morphology, the diagnosis is facilitated by family history in dominant conditions, whilst it is mainly based on biochemical and molecular findings in recessive and de novo cases. The typical examples of membrane defects are hereditary spherocytosis (HS), hereditary elliptocytosis (HE), and the group of hereditary stomatocytosis (Hst), which are mostly dominant [1, 2]. Table 1 shows the genetic basis of most common membrane defects. Glucose-6-phosphate dehydrogenase (G6PD) and pyruvate kinase (PK), are the most common enzyme deficiencies, showing an X-linked and recessive inheritance, respectively. Among rarer CHAs, it is worth mentioning several ultra-rare enzyme defects (glucosephosphate isomerase, GPI, phosphofructokinase, PFK, adenylate kinase, AK, triosephosphate isomerase, TPI, phosphoglycerate kinase, PGK, hexokinase, HK, and pyrimidine 5′-nucleotidase, P5N), and congenital dyserythropoietic anemias (CDA), mostly recessive conditions. Table 2 shows the genetic basis of the listed enzymatic defects and CDAs [3–7]. The diagnosis of CHAs is sometimes challenging, due to their rarity, their variable phenotype, and the need of specialized work-up and genetic testing. Moreover, several confounders should be considered, including concomitant blood loss, vitamin/iron deficiency, alterations of hemolytic markers due to other causes, coexistence of other hemolytic disorders, and association with bone marrow, renal or hepatic diseases. Figure 1 highlights the diagnostic flow chart of the most common CHAs, along with the most frequent confounding factors, and Table 3 illustrates the main diagnostic features. In this review we will provide some instructive clinical vignettes that highlight the difficulties and confounders encountered in the diagnosis and clinical management of CHAs.

**Table 1:** Genetic basis of red blood cell membrane defects

	Trasmission	Gene	Function
Hereditary spherocytosis (HS)	Autosomal recessive	*SPTA1*	Membrane skeletal network
	Autosomal dominant	*SPTB*	Membrane skeletal network
	Autosomal dominant	*SLC4A1*	Anion exchange channel Link to glycoltytic enzymes Vertical interactions
	Autosomal dominant, de novo	*ANK1*	Vertical interactions
	Autosomal recessive	*EPB42*	Stabilize band3/ankyrin complex
Hereditary elliptocyosis (HE)	Autosomal dominant	*SPTA1*	Membrane skeletal network
	Autosomal dominant	*SPTB*	Membrane skeletal network
	Autosomal dominant	*EPB41*	Stabilize spectrin-ankyrin contact
Hereditary pyropoikylocytosis	Autosomal recessive	*SPTA1/ SPTA1*^*LELY*^ *SPTA1/ SPTB* *SPTB/SPTB*	Membrane skeletal network
Hereditary stomatocytosis (HSt)			
Dehydrated	autosomal dominant	*PIEZO1*	Mechanosensitive ion channel
Overhydrated	autosomal dominant	*RHAG*	Rh -blood group
Gardos Channelopathy	autosomal dominant, de novo	*KCNN4*	Potassium Ca2 + -Activated Channel

**Table 2:** Genetic basis of red blood cell (RBC) enzymopathies and congenital dyserythropoietic anemia

	Trasmission	Gene	Function
*RBC enzyme defects*			
Glucose-6-phosphate Dehydrogenase deficiency (G6PD)	X-linked	*G6PD*	Hexose-monophosphate shunt
Pyruvate kinase deficiency (PKD)	Autosomal recessive	*PK-LR*	Glycolysis
Glucosephosphate isomerase deficiency (GPID)	Autosomal recessive	*GPI*	Glycolysis
Triosephosphate isomerase deficiency (TPID)	Autosomal recessive	*TPI1*	Glycolysis
Hexokinase deficiency (HKD)	Autosomal recessive	*HK1*	Glycolysis
Phosphofructokinase deficiency (PFKD)	Autosomal recessive	*PFK-M* *PFK-L*	Glycolysis
Phosphoglycerate kinase deficiency (PGKD)	x-linked	*PGK1*	Glycolysis
Pyrimidine-5’-nucleotidase deficiency (P5N)	Autosomal recessive	*NT5C3A*	Nucleotide metabolism
Adenylate kinase deficiency (AKD)	Autosomal recessive	*AK1*	Nucleotide metabolism
*Congenital dyserythropoietic anemias*			
CDA type I	Autosomal recessive	*CDAN1* *C15ORF41*	Microtubule attachments Restriction endonuclease
CDA type II	Autosomal recessive	*SEC23B*	Vescicle trafficking
CDA type III	Autosomal dominant	*KIF23*	Cytokinesis
CDA variants	x-linked	*GATA1*	Transcription factor
	Autosomal dominant	*KLF1*	Transcriptional activator

**Table 3:** Main diagnostic features of congenital hemolytic anemias (CHAS)

	Membrane defects	Enzyme defects	Congenital dyserythropoietic anemia
Anemia	Highly variable from severe to normal Hb values	Highly variable from severe to normal Hb values	Highly variable from very severe to nearly normal Hb values
Reticulocytes	Usually elevated except during aplastic crisis	Usually elevated except during aplastic crisis; particularly elevated in PKD after splenectomy	Usually low as referred to Hb values
Red cell morphology	Highly characteristic	Usually unremarkable	Usually unremarkable
Osmotic fragility tests, EMA binding, and ectacytometry	Typically altered	Usually uninformative	Usually uninformative
Membrane protein analysis	Informative in most cases	Useless	Usually uninformative, except for band 3 deglycosylation in CDA type II
Enzyme assays	Useless	Essential	Useless
Molecular tests	Informative particularly in HSt	Essential	Essential
Family history	Positive in most cases	Usually negative, consider consanguinity	Usually negative, consider consanguinity

Eosin 5 maleimide EMA binding; PKD pyruvate kynase deficiency; HSt hereditary stomatocytosis; CDA congenital dyserythropoietic anemia

## Hereditary spherocytosis (HS) and rarer membrane defects

HS is the most common CHAs in Caucasians, with an estimated prevalence ranging from 1–2/5000. Approximately 75% of cases display an autosomal dominant pattern of inheritance [1, 2, 8, 9]. The molecular defect involves the genes coding for RBC membrane proteins *ANK1* (ankyrin, 50–60% of HS patients), *SPTA1* or *SPTB* (α- or β-spectrin, 20% of HS, taken together), *SLC4A1* (band 3, 15–20% of cases), and *EPB42* (protein 4.2), the latter being mutated in autosomal recessive HS, more common in Japan but rarer in other populations (Table 1). In about 10% of HS it is not possible to identify any molecular basis for the disease [9]. A genotype–phenotype correlation may be drawn only to a certain extent in HS, with α-spectrin deficiency associated to more severe anemia and remaining mutations displaying a variable degree of anemia. These abnormalities result in loss of RBC membrane surface area, transformation of RBC from discocytes into spheroidal, osmotically fragile cells that are selectively destroyed in the spleen [1]. Clinically, variable degree of anemia, jaundice, splenomegaly and gallstones are the typical findings. Laboratory parameters include increased absolute number of reticulocytes, unconjugated hyperbilirubinemia, and lactate dehydrogenase (LDH), and reduced haptoglobin. The diagnosis is based on family history, RBC morphology, and on the increased osmotic fragility; further useful tests comprise flow cytometric test with eosin 5 maleimide (EMA binding), RBC deformability curve in ektacytometry, and study of membrane proteins in polyacrylamide gel (SDS-PAGE) [10, 11]. Molecular studies are usually not performed, since mutations in HS-related genes are dispersed and nonspecific as assessed by novel next generation sequencing studies [12, 13]. Given the heterogenous presentation, HS has been classified in classified in trait (normal Hb and reticulocytes, unconjugated bilirubin < 1 mg/dL), mild (Hb > 11 g/dL, reticulocytes 3–6%, unconjugated bilirubin 1- 2 mg/dL), moderate (Hb 8–12 g/dL, reticulocytes 6–10%, unconjugated bilirubin > 2 mg/dL) and severe (Hb > 8 g/dL, reticulocytes > 10%, unconjugated bilirubin > 3 mg/dL) [9]. Clinical management of HS mostly relies on folic acid supplementation, especially during infectious or surgical triggers, and splenectomy, when indicated according to the severity of anemia (see below). As for all other CHAs, intrinsically prone to gallstone formation, cholecystectomy is indicated in case of symptomatic lithiasis, and often performed concomitantly with splenectomy [14]. Finally, it is worth mentioning other rarer membrane defects such as HE and Hst that are diagnosed through the same work up suggested for HS, except for HSt that benefit from molecular evaluation. Specifically, HSt are a group of rare hemolytic anemias with autosomal dominant transmission, characterized by the inability to regulate the RBC cation homeostasis. Loss of cation content results in cell dehydration (dehydrated HSt), the most frequent form, caused by gain of function mutations in *PIEZO1* gene [15–17]. Other forms include overhydrated HSt, due to mutations in *RHAG* gene that cause an increase of intracellular water content [18] and the recently described Gardos channelopathy, due to *KCNN4* gene mutations that determine a modification of intracellular calcium concentration [16, 19] (Table 1).

### Clinical vignette 1: a typical clinical presentation of hereditary spherocytosis

A 53-year-old male presented with splenomegaly (19.3 cm at abdomen ultrasound) and altered hemolytic markers. Iron and vitamin levels were normal, no blood loss was documented, direct anti-globulin test (DAT), and hemoglobin (Hb) electrophoresis did not reveal pathological findings. Family history was unremarkable, and the subject had undergone cholecystectomy at the age of 27 years. The laboratory workup (RBC morphology, increased osmotic fragility, positive EMA binding, and ektacytometry) led to the diagnosis of HS trait (normal Hb, reticulocytes, and bilirubin), and the patient was followed, always displaying normal Hb levels. Six years later, Hb levels progressively worsened until 8.4 g/dL (requiring transfusion support) along with neutropenia (0.6 × 10^9^/L) and mild thrombocytopenia (120 × 10^9^/L). Reticulocyte counts were increased (200 × 10^9^/L) as well as unconjugated bilirubin (2.1 mg/dL) and haptoglobin was reduced. The DAT was again negative, as was the research of paroxysmal nocturnal hemoglobinuria (PNH) clone. Once excluded secondary causes (nutrients deficiencies, bleeding, chronic renal/liver diseases and inflammatory conditions), a bone marrow evaluation was performed. The latter showed no features of bone marrow failure/dysplasia, and the subject was vaccinated against capsulated bacteria before splenectomy.

### Comments to vignette 1

The clinical course depicted in this vignette is quite unusual as the severity of HS is generally observed during infancy and adolescence, concomitantly to infections and increased metabolic requests. Here, additional causes of hemolytic anemia and lack of bone marrow compensations were considered, as the subject was middle-aged. Bone marrow evaluation is not routinely performed in membrane and enzyme defects, but its involvement should be taken into account as it can be a confounder in the interpretation of the clinical picture and reduce the efficacy of splenectomy. The latter is usually of high effectiveness in HS, leading to a median Hb increase of 3 g/dL, associated with an amelioration of hemolytic markers [10, 14]. It is generally indicated in severe cases during adolescence (always after 5–6 years of age) and in case of symptomatic/painful splenomegaly. Additional findings to be considered are associated thrombocytopenia or leucopenia, and patient’s quality of life. For young adult subjects, unacceptable cutaneous jaundice and wish to become pregnant may balance the decision towards splenectomy. It is important to remember that splenectomy is accompanied by an infectious and thrombotic risk and has a greater risk in the elderly [20–23]. Thus, it must be preceded by anti-pneumococcal, anti-meningococcal, and anti-Haemophilus vaccine prophylaxis. Moreover, a prompt referral to the medical attention in case of suspected thrombosis or severe infections is mandatory. Of note, the possible existence of ectopic splenic tissue / accessory spleens may account for relapsing anemia after splenectomy.

### Clinical vignette 2: a case of HS and autoimmune hemolytic anemia (AIHA)

A 22-year-old female from Indian origin was referred to our center with a diagnosis of autoimmune hemolytic anemia (Hb 8.9 g/dL, LDH 2 × upper limit of normality, DAT positive for IgG) and increased spleen volume (19 cm at ultrasound). She had been treated with prednisone 1 mg/Kg day with partial response, followed by rituximab due to early relapse, again with partial response. At referral, a further Hb decrease was noted (8.5 g/dL) and splenectomy was considered. Given the young age, the presence of splenomegaly and gallstones, family history was reevaluated, although difficult to collect due to language barrier. A mild anemia was noted during adolescence and neonatal jaundice referred. Blood smear analysis revealed the presence of spherocytes which, although possibly present in AIHA, prompted the examination of a possible CHA. Osmotic fragility test and EMA-binding were consistent with a diagnosis of HS, but ektacytometry showed a pattern suspicious for dehydrated HSt. Therefore, mutational analysis for HSt genes was performed and returned negative. The patient underwent splenectomy and cholecystectomy with complete recovery of her anemia of both causes, autoimmune and congenital.

### Comment to vignette 2

Although AIHA is rarely observed together with CHAs due to membrane defects, several reports have been described of AIHA complicating congenital anemias, including thalassemia and sickle cell disease [24, 25]. Thus, DAT should always be evaluated not only at diagnosis, but also performed during the follow up, particularly in case of unexpected Hb drop. Therapy of AIHA includes steroids, rituximab, and splenectomy. Since the latter therapeutic option was adopted, the patient has been extensively studied to exclude a diagnosis of HSt, given the increased thrombotic risk observed in this condition after splenectomy, that is contraindicated particularly in the dehydrated form [14, 26–28]. The case also highlights that ektacytometry is pivotal in the differential diagnosis of membrane defects. However, it is worth mentioning that “secondary” spherocytosis is observed also in AIHA, due to partial phagocytosis of autoantibody-bound RBCs, so that ektacytometry pattern may be indistinguishable from HS.

## Pyruvate kinase deficiency (PKD)

PKD is the most frequent non-spherocytic CHA (estimated prevalence of 3–8 per 1,000,000) caused by autosomal recessive variants in the *PKLR* gene. PKD is highly heterogeneous from a biochemical, clinical and genetic point of view, since over 300 pathogenic mutations in the *PKLR* gene have been described so far [7]. The diagnosis should be suspected in the presence of clinical signs and symptoms and laboratory markers of chronic hemolytic anemia and is based on the demonstration of reduced PK enzymatic activity, and on the detection of compound heterozygous or homozygous mutations in the *PKLR* gene [29, 30]. Enzymatic activity may give falsely normal levels in the presence of an increased number of reticulocytes, recent transfusions, or incomplete removal of platelets or white blood cells. Thus, the diagnosis should be confirmed by *PKLR* genotyping: more common mutations are missense substitutions, while disruptive mutations (stop codons, frameshifts, and large deletions) are less frequent and generally associated with a more severe phenotype [7, 31]. Advantages of the genetic testing are the small blood volume required, no interference by transfused RBCs, and suitability for prenatal diagnosis. Disadvantages are the difficulty to identify large deletions and intronic mutations, and the interpretation of newly reported mutations as causative without functional tests. Therefore, both PK enzyme activity and *PKLR* genetic testing are recommended to confirm the diagnosis of PKD [29, 30]. Clinical presentation is highly variable, ranging from hydrops fetalis and prematurity, to fully compensated hemolysis (indirect hyperbilirubinemia or reticulocytosis) without anemia. Most subjects receive at least one transfusion, particularly during infancy/adolescence and generally during infectious episodes. About 20% of patients are transfusion-dependent, and contrarily to HS, splenectomy (performed in more than half of cases) only slightly ameliorates anemia (median rise in Hb of 1.6 g/dl). In addition, transfusion dependence persists in 10% of patients even after splenectomy [30, 31]. Bone marrow transplant has been only anecdotally reported, with high toxicity, and is not generally pursued. Experimental new treatments are in progress, including an oral activator of PK enzyme (mitapivat) and gene therapy [32, 33] (NCT04105166). Complications are frequent and include iron overload (48%), gallstones (45%), thrombosis (11%), and more rarely osteopenia, aplastic crisis, extramedullary hematopoiesis, endocrine disfunction, pulmonary hypertension and leg ulcers [31, 34, 35].

### Clinical vignette 3: a severe PKD with alloimmunization

A 19-year-old male had been diagnosed with severe PKD since infancy and regularly transfused (1 RBC unit every 1–2 months). Transfusion burden persisted even after splenectomy performed at 6 years of age. He was on regular chelation because of iron overload and suffered from several infections. He was referred due to increased transfusion need and drop of pre-transfusion Hb levels. An aplastic crisis was excluded given increased reticulocytes (typically observed in splenectomized PKD) and platelets, and negative infectious screening (Parvovirus B19, cytomegalovirus, Epstein-Barr virus, etc.). DAT and indirect anti-globulin test (IAT) were both positive and further investigation led to the identification of alloantibodies anti-Fy^a^.

### Comment to vignette 3

A possible complication of transfusions is the development of alloantibodies. The latter generally show an evanescent pattern so that only about 30% are usually detected. Their clinical significance is variable and largely depends on the amount of transfused RBCs and on their type and titer. The most common blood groups involved in alloimmunization are Rh, Kell, Duffy, and Kidd and the relative antigens (CDEec, KkJs^a^, Jk^a^Jk^b^, Fy^a^Fy^b^, Lu^a^Lu^b^, MNSs). These alloantibodies may cause increased hemolysis that might falsely be attributed to the underlying CHA and may result in augmented transfusion need. Alloantibodies should be excluded with absorption techniques and extended phenotyping/genotyping of the above cited antigens [36]. Genotyping is currently recognized as the preferred technique allowing the selection of the best antigen-matched units [37]. A good communication between the clinician and the transfusion center is advisable in transfusion dependent cases.

## Glucose-phosphate isomerase deficiency (GPID)

GPID is the second most common enzymopathy of glycolysis, following PKD. It usually causes mild to severe chronic hemolytic anemia and rarely intellectual disability or neuromuscular symptoms [6, 38–41]. GPID is transmitted as an autosomal recessive trait and gene locus is located on chromosome 19q13.1 [42]. About 60 patients with GPID have been described, and about 40 mutations have been reported so far. Missense mutations are the most common, but non-sense and splicing mutations have also been observed. A series of 12 subjects with GPID has been recently reported, showing a median age at diagnosis of 13 years (1–51), displaying moderate to severe anemia that improved with aging, and no neurological symptoms. Serum ferritin levels were increased in most patients and two of them required iron chelation. Six novel mutations in the GPI gene were identified and were considered pathogenic [6].

### Clinical vignette 4: A case of GPID with iron overload

A 42-year-old male was referred for chronic hemolytic anemia since infancy with a presumed diagnosis of congenital membrane defect and iron overload (ferritin levels 2346 ng/mL, and transferrin saturation of 89%). During childhood transfusion dependence was present and splenectomy was performed at 9 years of age obtaining transfusion independence. Moreover, cholecystectomy was performed at the age of 15 due to frequent abdominal pain. Family history was unremarkable, although parents might have been distant relatives (originating from a small village). Screening for membrane defects was normal, and enzymatic testing revealed a GPI deficiency (32% enzymatic residual activity), while other enzymes (G6PD, PK, PFK, AK, PGK, TPI, HK, and P5N) were normal. Molecular analysis confirmed the diagnosis, by showing two compound mutations. Testing for hereditary hemochromatosis gave negative results, and T2* MRI study showed a moderate iron overload (liver iron concentration > 4 mg Fe/g dry weight). Oral iron chelation was started with progressive amelioration of iron parameters and normalization within 1 year of therapy. Chelation was stopped and cyclically resumed, monitoring ferritin levels and transferrin saturation.

### Comment to vignette 4

Given the rarity of GPID, the diagnosis was delayed of several years and was reached only at a reference center where biochemical tests and molecular tools for rarer enzymopathies were available. The peculiarity of this case is the important iron overload, that may be observed in all CHAs, and that is partly explained by the transfusion dependence in severe anemic patients. Iron overload is poorly considered in CHAs, at variance with hemoglobinopathies and the possible coexistence of hereditary hemochromatosis should always been investigated. In fact, even a heterozygous state for the latter, combined with chronic hemolysis, may result in iron overload. A recent study evaluated cardiac and hepatic MRI in subjects with different CHAs and found iron overload found in 40% of patients. The association of ferritin > 500 μg/L plus transferrin saturation > 60% was demonstrated as the best combination to predict MRI findings. Iron overload was also associated with increased erythropoietin and hepcidin values and with augmented inflammatory cytokine levels, suggesting the existence of a vicious cycle between chronic hemolysis, inflammatory response and iron in CHAs [6]. Iron chelation and its monitoring are only partially defined in patients with CHAs and most clinicians rely on the experience of hemoglobinopathies. The monitoring of serum ferritin and transferrin saturation appears cost effective, whereas no clear indications for sequential MRI assessment are available [43]

## Triosephosphate isomerase deficiency (TPID)

Among ultra-rare enzyme deficiencies, it is worth mentioning a group of conditions where hemolysis is associated with several non-hematologic manifestations. The latter occur when the defective enzyme is not confined to the red cells but also expressed in other tissues, such as in TPI, PGK, PFK, and AK. Clinical features include neurologic impairment, myopathy, and frequent infections, which are variably combined in the different phenotypes. Most of these conditions display recessive inheritance, heterogeneous clinical presentation and severity, challenging the diagnosis. TPI deficient patients nearly always display serious neuromuscular disease and susceptibility to infections, and most of them die in the first decade of life [3, 44, 45].

### Clinical vignette 5: TPID and severe neurological involvement

A 2-year-old child suffered since birth from severe neurologic dysfunction, with encephalopathy, psychomotor impairment, neuropathy, apostural tetraparesis, and diaphragmatic paralysis. She suffered from several infectious episodes, had been subjected to tracheostomy for respiratory insufficiency, to gastrostomy to allow nutrition, and required occasional transfusion support for episodes of anemia. Investigations for congenital metabolic diseases included plasma, urine and cerebrospinal fluid amino acids, urine organic acids, plasma acylcarnitine profile, and urine mucopolysaccharides and oligosaccharides, all resulting within the normal range. Moreover, tests for congenital disorders of glycosylation, as well as bone marrow examination gave no insights into this complex presentation. Thereafter, attention was paid to persistent anemia, and some hemolytic features were noted, although difficult to interpret in the clinical context. At referral, Hb was 7.4 g/dL, and peripheral blood smear revealed the presence of dacrocytes, spherocytes, elliptocytes, schistocytes, and target cells. Osmotic fragility tests, EMA-binding and SDS analysis of membrane proteins were normal. Enzymatic activity tests displayed a marked reduction of TPI, whilst G6PD, PK, GPI, PFK, AK, PGK, HK, and P5N were within the normal range. Molecular studies confirmed the diagnosis of TPI deficiency, by showing a compound heterozygosity in the proband. The same mutations were present in the parents at heterozygous state.

### Comment to vignette 5

As also shown in the clinical vignette 4, the diagnosis of ultrarare enzymatic deficiencies relies on specialized analyses that are available only in few dedicated laboratories. In the case described, the diagnosis was particularly challenging given the extra-hematological manifestations that puzzled clinical picture. Overall, this vignette highlights the importance of deepening the diagnostic assessment of hemolytic features noted in individuals with syndromic phenotypes and extra-hematological features. Moreover, molecular characterization of the defect is important to confirm the diagnosis and to allow genetic counseling and prenatal diagnosis in more severe cases.

## Congenital dyserythropoietic anemia (CDA)

CDAs are a group of very rare congenital anemias (prevalence of 1–9 per 1,000,000) marked by ineffective erythropoiesis and morphological abnormalities of erythroblasts. Clinically, anemia and hemolytic features are variable, but usually characterized by inadequate reticulocytosis [5, 46–49]. CDAs classification include three major types (I, II and III) and other rarer variants or sporadic. The identification of these subtypes is based on the typical morphology of bone marrow erythroblasts and, more recently, on the detection of the characteristic mutations [50, 51]. The latter have also allowed to clarify some of the pathogenic mechanisms affecting cell maturation and division. CDA type I (CDAI), inherited as a recessive disorder, is caused by mutations in *CDAN1* (CDAIa) or *c15orf41* (CDAIb) genes. Bone marrow is characterized by the presence of binucleated erythroblasts, chromatin bridges between nuclei, and “Swiss cheese” appearance of dense heterochromatin at electron microscopy [52]. CDA type II (CDAII) is also inherited as a recessive disease, and is caused by mutations in the *SEC23B* gene [53, 54]. Membrane protein investigation demonstrates a typical hypoglycosylation of band 3, and bone marrow shows binucleated and multinucleated erythroblasts with a peripheral double membrane. CDA type III, differently from the previous forms, has a dominant inheritance, and is caused by mutation of *KIF23* [55]. Finally, rarer CDA variants are CDA type IV (associated with the dominantly inherited *KLF1* gene mutation) [56] and an X-linked sporadic form caused by *GATA 1* mutation [57] (Table 2).

### Clinical vignette 6: a case of CDAII and severe bleeding

A 36-year-old man was referred for moderate macrocytic anemia with inadequate reticulocytosis (Hb 9.8 g/dL, mean corpuscular volume 106 fL, 80 × 10^9^/L reticulocytes), mild hemolytic features (LDH 1.2 × upper limit of normality, unconjugated bilirubin 1.2 mg/dL, undetectable haptoglobin), and splenomegaly (18 cm by ultrasound). Diagnostic workup (negativity of osmotic fragility tests and ektacytometry, normal RBC enzymes assays, hypoglycosylation of band 3, bone marrow dyserythropoiesis, and detection of SEC23B mutation) led to the diagnosis of CDAII and the subject was put on clinical follow up. One year later, Hb levels progressively decreased to 6–7 g/dL requiring prompt transfusion. Additional causes of anemia were investigated with the identification of erosive gastro-duodenitis with chronic/subacute blood loss. Treatment with proton pump inhibitors led to gastro-duodenitis amelioration and Hb levels progressively stabilized at about 8 g/dL. Given the progressive increase of spleen size (21 cm diameter) and overall poor quality of life, the patient has been splenectomized obtaining stable Hb levels between 10.5 and 11.5 g/dL.

### Comment to vignette 6

This is a typical case with a defined diagnosis of CHA in which a superimposed cause of anemia challenged clinical management. Blood loss is usually accompanied by compensatory reticulocytosis that was not observed in this case due to bone marrow dyserythropoiesis. Moreover, transfusions may also jeopardize the evaluation of bone marrow compensation. Once identified and corrected the blood loss, there was a treatment need for CDAII. Data from literature report that splenectomy is not as effective as in HS, leading to an average Hb increase of about 1 g/dL [10, 14, 58]. Given the well-known infectious and thrombotic complications of splenectomy, a clear indication is not easy to establish. Recent recommendations from the European Hematology Association state that splenectomy in CDAII should be considered in severely anemic cases and/or in those with symptomatic splenomegaly [14]. The only curative treatment for severe CDA is hematopoietic stem-cell transplantation, which has been reported only in few pediatric cases with good outcome [59]. Potential future therapies include drugs targeting ineffective erythropoiesis such as sotatercept and luspatercept, that have been shown effective in CDAII murine models, and in thalassemia and myelodysplastic syndromes [60–62].

## Undiagnosed CHAs

Despite extensive and complete morphologic, biochemical, and molecular investigation, about 20% of CHAs remain undiagnosed. This results in a disappointing burden of consultations and tests for the individuals, disproportional resource utilization, risk of inappropriate therapies such as splenectomy, and preclusion of novel specific treatments such as mitapivat in PKD. The recent availability of next generation sequencing (NGS) technologies has greatly improved the diagnostic approach to CHAs. However, the performance of this technique largely depends on the NGS strategies adopted, including different targeted panels or whole exome sequencing (WES) [63]. The latter are not routinely available and their interpretation still relies on clinical findings.

### Clinical vignette 7: a man with undiagnosed CHAs and Gaucher disease

A 31-year-old male was referred for mild pancytopenia (Hb 11 g/dL, PLT 62 × 10^9^/L, WBC 2.3 × 10^9^/L), splenomegaly (16 cm diameter), and hyperferritinemia (620 ng/mL). These features were present since adolescence, and he had received several consultations and undergone countless investigations without a definite diagnosis. Extensive work up for CHAs (morphology, study of membrane proteins, erythrocyte enzymes, EMA-binding, ectacytometry, and NGS panel) resulted negative. By re-examining the clinical history, a recently implemented algorithm for the diagnosis of Gaucher disease (GD) was considered. The latter relied on two main criteria, i.e. splenomegaly and/or thrombocytopenia associated with at least one among the following: bone pain history, anemia, monoclonal gammopathy of unknown significance, polyclonal gammopathy in subjects under 30 years of age and splenectomy. The subject underwent testing for β-glucosidase enzyme activity on dried blood spot, resulting positive. The positivity was confirmed by specific genetic analysis (GBA1 gene mutation) and led to the diagnosis of GD.

### Comment to vignette 7

Although splenomegaly is one of the most common characteristics of CHAs, there are many other causes that should be taken into account. They may be classified according to the pathogenic mechanism including increased spleen function, abnormal blood flow, and spleen infiltration. The first group includes CHAs, infections (mononucleosis, viral hepatitis, splenic abscess, typhoid fever, brucellosis, leptospirosis, tuberculosis, histoplasmosis, malaria, leishmaniasis, trypanosomiasis), autoimmune diseases (rheumatoid arthritis, systemic lupus erythematosus, etc.), and extramedullary hematopoiesis. The vascular causes encompass hepatic hypertension (cirrhosis, Budd–Chiari syndrome), and hepatic schistosomiasis and echinococcosis. Finally, infiltrative diseases include neoplastic diseases (leukemias, lymphomas, methastatic solid tumors) and the rare metabolic disorders (GD, Niemann–Pick disease, alpha-mannosidosis, Hurler syndrome and other mucopolysaccharidoses, amyloidosis, and Tangier disease). Despite this broad list, the diagnosis of GD is quite easy provided clinical suspicion. GD is the most common lysosomal hereditary disorder due to the deficiency of the β‐glucosidase enzyme causing the accumulation of glucosylceramide in the reticuloendothelial cells. It is an autosomal recessive disorder with an elevated prevalence in the Ashkenazi Jewish population (1/600, carrier rate 1/15) compared to the non‐Ashkenazi population (1/75000 births). The application of the above cited diagnostic algorithm led to the diagnosis of GD in 7 out of 196 previously undiagnosed patients, allowing substitutive enzymatic therapy [64, 65].

## Conclusions

The diagnosis of CHAs may be challenging due to their rarity, poor knowledge, and to the requirement of specialized diagnostic work-up and genetic testing. Additional difficulties reside in their heterogeneous clinical phenotype, which may include extra-hematologic and neurologic findings, causing referrals to different specialists. This may lead to a delayed diagnosis, with a burden of consultations and tests for the patient, and aconsequent inappropriate healthcare resource utilization. Moreover, incorrect diagnoses may cause inappropriate therapies such as splenectomy in HSt and missed-diagnoses may prevent the access to novel specific treatments such as mitapivat for PKD, or drugs targeting ineffective erythropoiesis in CDAs. New molecular tools, such as NGS and WES, would greatly improve the diagnostic gaps in the near future, provided their broader availability and critical clinical interpretation. Additionally, they can be of great value for genetic counseling, which is increasingly asked by affected families. More importantly, several even common confounders should be considered, as highlighted in the clinical vignettes presented. They include the possible coexistence of other diseases, such as mild myelodysplasia in advanced age, which may hamper the bone marrow compensation, or a banal and underestimated blood loss. DAT-positivity due to alloimmunization, which is a fairly common and known finding in hemoglobinopaties, should always be considered in transfusion-dependent CHAs, as well as the coexistence of a true DAT-positive autoimmune hemolytic anemia. Likewise, it is worth considering the presence of hemolysis associated with mechanical injury, toxic agents, and infections, or of small paroxysmal nocturnal hemoglobinuria clones, that may be found in healthy subjects and in several other hematologic diseases [66, 67]. Iron overload is a frequent finding in CHAs, and this underestimated complication may be the main reason for referral. Likewise, isolated splenomegaly may be a reason for referral to the hematologist, leading to investigate boundless infectious, autoimmune and lymphoproliferative/neoplastic conditions to unravel the differential diagnosis. Finally, when an extensive investigation is inconclusive, even diagnoses completely different from CHAs should be taken into account. These include one of the several metabolic disorders, such as GD illustrated in the last vignette. Overall, confounders should always be considered, keeping an open-mind attitude across the several congenital and acquired diseases with hemolytic features, and maintaining a tight interaction between clinicians and laboratory researchers.

## Data Availability

Not applicable.
